# Estimating age-stratified transmission and reproduction numbers during the early exponential phase of an epidemic: A case study with COVID-19 data

**DOI:** 10.1016/j.epidem.2023.100714

**Published:** 2023-08-15

**Authors:** Zachary Stanke, John L. Spouge

**Affiliations:** National Center for Biotechnology Information, National Library of Medicine, National Institutes of Health, Bethesda, MD 20894, USA

**Keywords:** COVID-19, Asymptomatic transmission, Next-generation matrix (NGM), Transmission per contact, Prem contact matrix

## Abstract

In a pending pandemic, early knowledge of age-specific disease parameters, e.g., susceptibility, infectivity, and the clinical fraction (the fraction of infections coming to clinical attention), supports targeted public health responses like school closures or sequestration of the elderly. The earlier the knowledge, the more useful it is, so the present article examines an early phase of many epidemics, exponential growth. Using age-stratified COVID-19 case counts collected in Canada, China, Israel, Italy, the Netherlands, and the United Kingdom before April 23, 2020, we present a linear analysis of the exponential phase that attempts to estimate the age-specific disease parameters given above. Some combinations of the parameters can be estimated by requiring that they change smoothly with age. The estimation yielded: (1) the case susceptibility, defined for each age-group as the product of susceptibility to infection and the clinical fraction; (2) the mean number of transmissions of infection per contact within each age-group; and (3) the reproduction number of infection within each age-group, i.e., the diagonal of the age-stratified next-generation matrix. Our restriction to data from the exponential phase indicates the combinations of epidemic parameters that are intrinsically easiest to estimate with early age-stratified case counts. For example, conclusions concerning the age-dependence of case susceptibility appeared more robust than corresponding conclusions about infectivity. Generally, the analysis produced some results consistent with conclusions confirmed much later in the COVID-19 pandemic. Notably, our analysis showed that in some countries, the reproduction number of infection within the half-decade 70–75 was unusually large compared to other half-decades. Our analysis therefore could have anticipated that without countermeasures, COVID-19 would spread rapidly once seeded in homes for the elderly.

## 1. Introduction

Many epidemic models are elaborations of the idealized Susceptible-Infected-Recovered (SIR) model of (Kermack and McKendrick, 1927, 1932, 1933). Most of these epidemic models have at least three distinctive phases: a beginning, middle, and end (Spouge, 2021). The beginning of the epidemic displays exponential growth exp(*rt*), where r is an exponential growth rate, a constant reflecting epidemic penetration into the initial steady state of a population. The middle of the epidemic starts when dynamic variables like depletion of susceptibles and societal responses cause noticeable departures from the steady state. At the end, the epidemic burns itself out, and the population enters a final steady state. Sometimes, the beginning of the epidemic helps predict the final steady state (Ball and Clancy, 1993; Ma and Earn, 2006; Miller, 2012). Some epidemics begin with a sub-exponential phase (Chowell et al., 2019), so the present article excludes them from consideration.

Social contacts between different age-groups at home, work, school, or elsewhere help shape the epidemic spread (Prem et al., 2017). In principle, knowledge of age-specific epidemic parameters permits individuals or governments to modify social behaviors specifically and rationally (Koelle et al., 2022) to reduce both consequences of the epidemic and repercussions from the countermeasures (Lewis, 2022). Unfortunately, data for modeling and parameter estimation are sparsest at the beginning of an epidemic when knowledge of the infectious parameters could have the greatest impact.

At the beginning of the COVID-19 epidemic in 2020, e.g., investigators recognized that the epidemic spread in different age-groups had great relevance to herd immunity (e.g., Britton et al., 2020; Gomes et al., 2020) and to non-pharmaceutical interventions (NPIs) like school closures, lockdowns, etc. (e.g., Flaxman et al., 2020; Katul et al., 2020). Empirically, children appeared to spread COVID-19 (Forbes et al., 2020) but were less susceptible to it (Hippich et al., 2021; Viner et al., 2021; Vogel, 2020), causing conclusions about the importance of school closures to epidemic control to conflict (Levinson et al., 2020; Snape and Viner, 2020).

Of particular interest, (Davies et al., 2020) estimated age-specific epidemic parameters from data in six countries. Some of their data went beyond the exponential phase to include NPIs. The Prem matrices yielded the age-specific contact rates at home, work, school, or elsewhere for the exponential phase. Linear manipulations then extended the contact matrices for use during NPIs (Prem et al., 2017).

During NPIs, however, the population deviates from its initial steady state, complicating the biological interpretation of estimated parameters. In addition, individuals within different societies adapt idiosyncratically to NPIs (Corpuz, 2021), e.g., so ad hoc corrections to the Prem matrices introduce additional approximations logically far removed from the original data collected for the matrices in the steady state. Notably also, (Davies et al., 2020) estimated some individual age-specific parameters less accurately than some of their algebraic combinations. All these issues raise questions whose answers could aid the initial navigation of future epidemics. During the exponential phase, e.g., which parameters and parameter combinations are statistically identifiable? What difficulties are intrinsic to studies early in an epidemic?

This article is structured as follows. Our Methods and Materials section elaborates on the linear analysis in (Nishiura et al., 2010), who used maximum likelihood methods to examine age-specific susceptibility during a Japanese influenza epidemic. Our model of COVID-19 adds two features: (1) the undetected infections that dogged COVID-19 parameter estimation (Lavezzo and al., 2020) and (2) the question of age-specific infectivity (Han et al., 2021). The model deliberately mirrors the ground state of epidemiological ignorance about susceptibility, infectivity, and asymptomatic infection that pertained before the specifics governing COVID-19 became known. For each of the six countries studied, the Results section uses age-specific case data made available publicly before April 23, 2020, near the end of the exponential phase of COVID-19 in the countries examined. It successfully estimates the diagonal of both the age-stratified next-generation matrix (NGM, a common acronym (Diekmann et al., 2013), p.163) and the matrix of mean transmissions per effective contact, yielding the reproduction numbers and mean transmissions per contact within each age-group. Although COVID-19 displayed susceptibilities, infectivities, and clinical fractions that all varied with age, other epidemics may not display age-variation for all three parameters. For such epidemics, a reduced model holding one of the three parameters constant across age-groups becomes appropriate. For the three reduced models (the first two appearing elsewhere, in Davies et al., 2020), our methods yield the complete age-stratified next-generation and matrices of mean transmissions per contact. The Discussion examines our model assumptions, biases, and implications for future work.

Sections and tables within “Supplementary Information.docx” in the SI are preceded by “S”, e.g., Section S1 and Table S1. Rather than explicitly and repeatedly referring the reader to the SI for mathematical details, most sections within the SI correspond directly to sections in the main text and have the same numbers except for the prefix “S”. Other sections within the SI have a postfix “a”, e.g., S2.1a. Below, sections, figures, and tables without the prefix “S” refer to the main article.

## 2. Methods and materials

### 2.1. Theory

Six countries provided our data. Until Section 2.2, however, we consider only a single country.

Let a index the rows of a matrix; b, its columns. For example, let $C=∥c_{a,b}∥$ denote a matrix; $u=\left[u_{a}\right]$, a column vector. Our model starts with the Prem contact matrix $C=∥c_{a,b}∥$ for a single country, where $c_{a,b}$ gives the rate (in contacts/day) at which an individual in Stratum b (i.e., age-group b) contacts individuals in Stratum a, potentially infecting them (Prem et al., 2017, p.6) (the direction of epidemiological contact does not accord with matrix conventions in all fields). (Prem et al., 2017) note that C may be asymmetric, e.g., a child may breathe on a parent more frequently than vice versa. The Prem matrix C stratifies the population in the country by half-decades of life (0–4, 5–9, etc.) up to age 79, making C a 16×16 matrix (a,b=1,2,.,16). Consequently, unless specified otherwise, matrices below are 16×16; column vectors, 16×1.

The contact rate $c_{a,b}$ is related to (but different from) the reproduction number $r_{a,b}$, defined as follows. In a completely susceptible population, $r_{a,b}$ is the average number of individuals in Stratum a infected by a single typical infected individual in Stratum b during the individual’s entire infectious period (not contacts per day). Let “:= ” denote a definition. As usual, define the next-generation matrix (NGM) $R≔∥r_{a,b}∥$ (e. g., Kinoshita and Nishiura, 2017; Nishiura et al., 2010). In conjunction with quantities corresponding to susceptibility and infectivity, C yields R, as follows.

Let individuals in Stratum a have susceptibility $s_{a}\geq 0$ to infection (i. e., individuals in some strata may be more readily infected than in others, increasing $s_{a}$ (Nishiura et al., 2010) and (viral) infectivity $v_{a}\geq 0$ (e.g., individuals in some strata may exhale more virus than in others, increasing $v_{a}$). Infectivity $v_{a}$ is proportional to the force of infection per infection from Stratum a. Define the corresponding column vectors $s≔\left[s_{a}\right]$ and $v≔\left[v_{a}\right]$. Some authors define susceptibility as the probability of infection given contact (e.g., Davies et al., 2020), but our article treats both susceptibility and infectivity as relative, a ratio to a standard (e.g., as in Nishiura et al., 2010). Specifically, Section 2.2 uses the third decade 20–29 of life as the arbitrary standard.

Early in an epidemic, knowledge about infection and its time-course in different subpopulations is scarce. Ignorance suggests the expedient of assuming that susceptibility $s_{a}$ and infectivity $v_{a}$ are approximately constant throughout the infectious period, and that the infectious period in every stratum has the same mean duration ($\tau_{I}$, in days).

Given a column vector $x≔\left[x_{a}\right]$, define the diagonal matrix diag$(x)\;:\;=diag\left[x_{a}\right]=∥x_{a,b}∥$, where $x_{a,b}=x_{a}$ if a=b and $x_{a,b}=0$ otherwise. In a susceptible population with very few infections, the mean number of infections in Stratum a caused by a typical infected individual in Stratum b is proportional to the product of the infectivity $\left(v_{b}\right)$, the contact rate $\left(c_{a,b}\right)$, and the susceptibility $\left(s_{a}\right):r_{a,b}=s_{a}c_{a,b}v_{b}/\alpha$, where α is a proportionality constant dependent on the mean duration $\tau_{I}$ of the infectious period. Define the 16×16 diagonal matrices S≔diag⁡(s) and V ≔diag⁡(v), so the NGM

(1) $$R=\left\|r_{a,b}\right\|=\left\|s_{a}c_{a,b}v_{b}\right\|/\alpha =SCV/\alpha$$

(Kinoshita and Nishiura, 2017; Nishiura et al., 2010), where the overall proportionality constant α (in units of 1/days) effectively absorbs all proportionality constants in individual factors of **SCV**.

To sharpen the biological interpretation of $s_{a}$ and $v_{a}$ (e.g., see Nishiura et al., 2010), note that $r_{a,b}=s_{a}c_{a,b}v_{b}/\alpha =\left[s_{a}v_{b}/\left(\alpha \tau_{I}\right)\right]\left(c_{a,b}\tau_{I}\right)$. Because $c_{a,b}\tau_{I}$ is the mean number of contacts to Stratum a from an individual in Stratum b during the infectious period, $t_{a,b}=s_{a}v_{b}/\left(\alpha \tau_{I}\right)$ is mean number of infections in Stratum a transmitted per contact from a typical infected individual in Stratum b. Define the matrix of transmission per contact (more precisely, the matrix of mean transmissions per contact)

(2) $$T≔\left\|t_{a,b}\right\|=\left\|s_{a}v_{b}\right\|/\left(\alpha \tau_{I}\right)$$

The quantity $t_{a,b}$ derives its operational definition from the term “contact” for the Prem matrices (Prem et al., 2017). If transmission requires a contact and never occurs without it, $t_{a,b}$ is the transmission probability per contact. If, however, each infectious contact (as defined in Prem et al., 2017) corresponds on average to more than one infection, e.g., then $t_{a,b}>1$.

If the initial exponential growth of an epidemic continues for a sufficiently long time, the distribution of infections among the strata eventually approaches a limiting equilibrium probability distribution $\text{u}\;:\;=\left[u_{a}\right]$ (Nishiura et al., 2010), where $u_{a}$ represents the probability of a random infection being in Stratum $a\left(\sum u_{a}=1\right)$. Then,

(3) $$\left(SCV/\alpha \right)u=Ru=R_{0}u$$

where $R_{0}$ is the basic reproduction number for the country’s entire population (Diekmann et al., 1990; e.g., see also Diekmann et al., 2010; van den Driessche and Watmough, 2002; Wallinga and Lipsitch, 2007).

Because of asymptomatic infections during the COVID-19 epidemic, the recorded case count excluded many infections (Lavezzo and al., 2020). Call an infection that increases the case count “clinical”; otherwise, it is “subclinical”. On one hand, the term “subclinical” thereby gains an operational definition, though (as usual in biology) an imprecise one. On the other hand, a clinical infection is just a case. (Nishiura et al., 2010), e.g., essentially gave Eq. (3), which can handle data for diseases that spread exclusively through cases. The following develops the analogous analysis for diseases that spread through both subclinical infections and cases, e.g., COVID-19.

Assume that infections independently become cases with the probability ${\tilde{p}}_{a}$ (over-tildes here and below correspond to overt clinical infection), with $\tilde{P}≔diag\left[{\tilde{p}}_{a}\right]$. (Davies et al., 2020) call ${\tilde{p}}_{a}$ a “clinical fraction”. The fraction of cases in Stratum a is ${\tilde{u}}_{a}={\tilde{p}}_{a}u_{a}/\tilde{\alpha }$, where $\tilde{\alpha }$ normalizes ${\tilde{u}}_{a}$ to a probability distribution. Thus, $\tilde{u}≔\left[{\tilde{u}}_{a}\right]=\tilde{P}u/\tilde{\alpha }$. Matrix manipulation of Eq. (3) yields

(4) $$R_{0}\tilde{u}=R_{0}\left(\tilde{P}u/\tilde{\alpha }\right)=\tilde{P}\left(R_{0}u\right)/\tilde{\alpha }=\tilde{P}\left(Ru\right)/\tilde{\alpha }=\left(\tilde{P}R{\tilde{P}}^{-1}\right)\left(\tilde{P}u/\tilde{\alpha }\right)=\tilde{R}\tilde{u}$$

where $\tilde{R}≔\tilde{P}R{\tilde{P}}^{-1}$ is called the case-to-case NGM. Let $\tilde{R}=∥{\tilde{r}}_{a,b}∥$. If successive generations of infection have the same age-specific clinical fraction, then the case-to-case NGM $\tilde{R}$ has the following interpretation. In a susceptible population with very few infections, the case-to-case reproduction number ${\tilde{r}}_{a,b}≔{\tilde{p}}_{a}r_{a,b}{\tilde{p}}_{b}^{-1}$ counts the average number of cases in Stratum a after one generation of infection for each typical case in Stratum b. Eq. (3) for the NGM R reflects a causal relationship between infections in successive generations. In contrast, because spread from subclinical infection can cause a case, Eq. (4) for $\tilde{R}$ expresses a quantitative relationship between average case counts in successive generations, and it is not causal.

Eq. (5) below is central to the present article, and it requires the following formal definitions. Define the column vector of case susceptibilities $\tilde{s}=\left[{\tilde{s}}_{a}\right]=\left[{\tilde{p}}_{a}s_{a}\right]$, where ${\tilde{s}}_{a}$ is the susceptibility to being a case in Stratum a; and the column vector of augmented infectivities per case $\tilde{v}=∥{\tilde{v}}_{a}∥=∥v_{a}/{\tilde{p}}_{a}∥$, where ${\tilde{v}}_{a}$ is the infectivity of a case in Stratum a, after considering that the case’s spread is augmented by the spread from subclinical infections in its stratum. As with ${\tilde{p}}_{a}$, the over-tildes are a reminder that the quantities pertain to cases, not infections. Like the contrast between Eq. (3) for R and Eq. (4) for $\tilde{R}$, the definition of ${\tilde{s}}_{a}$ relies on a causal relationship, but the definition of ${\tilde{v}}_{b}$ is a (non-causal) relationship between averages. The corresponding diagonal matrices are $\tilde{S\;}≔diag(\tilde{s})$ and $\tilde{V}≔diag(\tilde{v})$, so they satisfy $\tilde{S}=\tilde{P}S$ and $\tilde{V}=V{\tilde{P}}^{-1}$.

Eq. (3) then yields

(5) $$\tilde{R}=\tilde{P}R{\tilde{P}}^{-1}=\tilde{P}\left(SCV/\alpha \right){\tilde{P}}^{-1}=\left(\tilde{P}S\right)C\left(V{\tilde{P}}^{-1}\right)/\alpha =\tilde{S}C\tilde{V}/\alpha$$

Eq. (2) also has an analog $\tilde{T}≔∥{\tilde{t}}_{a,b}∥$. The case-to-case matrix of transmission per contact

(6) $$\tilde{T}:=\tilde{P}T{\tilde{P}}^{-1}=\tilde{P}\left\|s_{a}v_{b}/\left(\alpha \tau_{I}\right)\right\|{\tilde{P}}^{-1}=\left\|\left({\tilde{p}}_{a}s_{a}\right)\left(v_{b}/{\tilde{p}}_{b}\right)/\left(\alpha \tau_{I}\right)\right\|=\left\|{\tilde{s}}_{a}{\tilde{v}}_{b}\right\|/\left(\alpha \tau_{I}\right)$$

where ${\tilde{s}}_{a}{\tilde{v}}_{b}/\left(\alpha \tau_{I}\right)$ is the mean cases in Stratum a per contact with a case in Stratum b (augmented again by the spread from subclinical infections in the same stratum).

Section 2.2 estimates $\tilde{s}=\left[{\tilde{s}}_{a}\right]=\left[{\tilde{p}}_{a}s_{a}\right]$ and $\tilde{v}=∥{\tilde{v}}_{a}∥=∥v_{a}/{\tilde{p}}_{a}∥$ from case counts alone. Unfortunately, the (infection-to-infection) NGM R and matrix T of transmission per contact are more biologically important than their case-to-case counterparts $\tilde{R}=\tilde{P}R{\tilde{P}}^{-1}$ and $\tilde{T}=\tilde{P}T{\tilde{P}}^{-1}$. The equations ${\tilde{r}}_{a,b}=\left({\tilde{p}}_{a}/{\tilde{p}}_{b}\right)r_{a,b}$ and ${\tilde{t}}_{a,b}=\left({\tilde{p}}_{a}/{\tilde{p}}_{b}\right)t_{a,b}$ show, however, that subclinical infections do not influence the diagonal elements ${\tilde{r}}_{a,a}=r_{a,a}$ and ${\tilde{t}}_{a,a}=t_{a,a}$ in R and T.

### 2.2. Data fit

Our study used age-stratified case counts for six countries: (1) Canada (2020); (2) China (Deng et al., 2020); (3) United Kingdom (2020); (4) Israel (2020); (5) Italy (2020); and (6) Netherlands (2020). In these six countries: (1) community spread dominated their initial cases (by contrast, e.g., many initial cases were imported in Norway); (2) they stratified their case data by decades; and (3) subject to Restrictions (1) and (2), their COVID-19 cases displayed the steepest initial exponential growth (Spouge, 2021). The cut-off at six countries is somewhat arbitrary but reflects difficulties demarcating the exponential phase of an epidemic if it does not contrast steeply and sharply with later phases.

Countries are indicated by parenthesized superscripts k=1,.,K(K=6). On one hand, $C=C^{\left(k\right)},R_{0}=R_{0}^{\left(k\right)},\tilde{R}={\tilde{R}}^{\left(k\right)}$ and $\tilde{u}={\tilde{u}}^{\left(k\right)}$ from Section 2.1 are superscripted, because they depend on the country k. We assume $C^{\left(k\right)}$ (Prem et al., 2017) and $R_{0}^{\left(k\right)}$ (Spouge, 2021) (Table S2) are known exactly for every country (or are at least less noisy than other calculations). On the other hand, we assume that the disease parameters $\tau_{I},\tilde{p},\tilde{s}$, and $\tilde{v}$ are common to all countries, so they lack superscripts. Implicitly, therefore, we are assuming that subclinical infections and cases are defined similarly in all countries. In principle, disease parameters derived purely from $\tau_{I},\tilde{p},\tilde{s}$, and $\tilde{v}$, e.g., ${\tilde{T}}^{\left(k\right)}$ and therefore $\alpha =\alpha^{\left(k\right)}$ do not depend on the country k. The Results check the accuracy of our assumptions by examining $\alpha =\alpha^{\left(k\right)}$ for approximate constancy across countries.

The Prem matrices omit over-80 age-groups, so our analysis had to omit the corresponding case data, with consequences addressed in the Discussion. Our analysis therefore considered only stratified case data for the first m=8 decades of life, i.e., the decades 0–9, 10–19, …, 70–79. Our study fit the case data, taking the infectious period $\tau_{I}$ in Table S1 and the basic reproduction numbers $R_{0}^{\left(k\right)}(k=1,.,K)$ in Table S2.

(Spouge, 2021) used the ARRP program (Park et al., 2005) to fulfill the need described in (Nishiura et al., 2010) for the “determination of an appropriate length of the exponential growth period”. Just as a Markov chain simulation requires a burn-in time to equilibrate, so an epidemic requires some unknown burn-in time during its exponential phase to attain its equilibrium age-distribution ${\tilde{u}}^{\left(k\right)}$. For each Country k, therefore, our age-stratified case counts came from a single health report, usually a two-weekly summary, near the end of the exponential phase. Every Country k reported a rapidly increasing number of COVID-19 cases over time, so the use of a single report excluded only a few cases in prior reports from consideration.

The remainder of the Methods gives an overview for fitting the parameters $\theta =(\tilde{s},\tilde{v})$.

Intuitively, each Prem matrix $C^{\left(k\right)}$ with the parameters $\theta =(\tilde{s},\tilde{v})$ determines a case-to-case NGM ${\tilde{R}}^{\left(k\right)}(\theta )={\tilde{R}}^{\left(k\right)}=\tilde{S}C^{\left(k\right)}\tilde{V}/\alpha^{\left(k\right)}$. On one hand, the computations retained 16 dimensions, to leverage the full half-decade resolution of the Prem matrix. On the other hand, the parameter constraints ${\tilde{s}}_{2i-1}={\tilde{s}}_{2i}$ and ${\tilde{v}}_{2i-1}={\tilde{v}}_{2i}(i=1,2,.,m$ for m=8) imposed the decade resolution of the case data on the half-decade resolution of $\theta =(\tilde{s},\tilde{v})$. The fit also required an arbitrary stratum for normalizing each of $\tilde{s}$ and $\tilde{v}$, so we arbitrarily chose the third decade $20-29:{\tilde{s}}_{5}={\tilde{s}}_{6}=1$ and ${\tilde{v}}_{5}={\tilde{v}}_{6}=1$. Results were not sensitive to the decade chosen for normalization. Thus, $\theta =(\tilde{s},\tilde{v})$ contains 2(8-1)=2(m-1)=14 free parameters.

The eigenvectors $\left[{\tilde{u}}_{a}^{\left(k\right)}(\theta )\right]$ of the matrix ${\tilde{R}}^{\left(k\right)}(\theta )$ have half-decade resolution, with dimension 16. To fit at the decade resolution of the case data, ${\tilde{u}}_{i,•}^{\left(k\right)}(\theta )≔{\tilde{u}}_{2i-1}^{\left(k\right)}(\theta )+{\tilde{u}}_{2i}^{\left(k\right)}(\theta )$ gives the equilibrium probability that a case falls into the i-th decade (i=1,2,.,m).

Under a Poisson model, a maximum likelihood estimate of the equilibrium probability ${\tilde{u}}_{i,•}^{\left(k\right)}(\theta )$ can fit $\theta =(\tilde{s},\tilde{v})$ to age-stratified case counts, as in (Nishiura et al., 2010) (Section 2.2.a in the SI gives details). Unfortunately, the maximum likelihood estimate was extremely sensitive to random noise, so we resorted to a regularized chi-square estimate of $\theta =(\tilde{s},\tilde{v})$, as follows.

### 2.3. Data fit with regularization

From the single health report from Country k(k=1,2,.,K for K=6 countries), let ${\tilde{n}}_{i,•}^{\left(k\right)}$ be the age-stratified case counts for the i-th decade of life (i=1,.,m), with total ${\tilde{n}}_{•,•}^{\left(k\right)}=\sum_{i=1}^{m}\;{\tilde{n}}_{i,•}^{\left(k\right)}$. The empirical fraction of cases in the i-th decade is therefore ${\hat{u}}_{i,•}^{\left(k\right)}≔{\tilde{n}}_{i,•}^{\left(k\right)}/{\tilde{n}}_{•,•}^{\left(k\right)}$.

Our fit minimizes the chi-square

(7) $$\chi^{2}\left(\hat{u}∥\theta \right)≔\sum_{k=1}^{K}n_{•,•}^{\left(k\right)}\sum_{i=1}^{m}\frac{{\left[{\hat{u}}_{i,•}^{\left(k\right)}-{\tilde{u}}_{i,•}^{\left(k\right)}\left(\theta \right)\right]}^{2}}{{\tilde{u}}_{i,•}^{\left(k\right)}\left(\theta \right)}$$

Section 2.3a justifies transforming the maximum likelihood fit into a chi-square fit.

A Tikhonov-Miller regularized fit (Press et al., 1976, p.808) reduces the sensitivity to noise by minimizing Eq. (7), but only after adding a penalty term for smoothness to prevent the coordinates of $\theta =(\tilde{s},\tilde{v})$ from oscillating too much as functions of age (see Section S2.3 in the SI for details). The penalty term reflects a biological intuition, that case susceptibility ${\tilde{s}}_{a}$ and augmented infectivity per case ${\tilde{v}}_{a}$ should not oscillate too wildly as decades of life increase.

Each country k=1,2,.,K (K=6) contributes a single constraint $\sum_{i=1}^{m}\;{\hat{u}}_{i,•}^{\left(k\right)}=1$ on the fractions of cases within each of m=8 decades of life that our fit considers. Eq. (7) therefore has an approximate chi-square distribution with N≔K(m-1)=6(8-1)=42 degrees of freedom. Thus, $\chi^{2}(\hat{u}∥\theta )$ has mean 42; variance, 84=2*42.

Our full model contains three parameters $\left(\tilde{p},s,v\right)$, but our statistical methods determine only the two parameters $\theta =(\tilde{s},\tilde{v})$, enough to estimate the case-to-case NGM ${\tilde{R}}^{\left(k\right)}=∥{\tilde{s}}_{a}c_{a,b}^{\left(k\right)}{\tilde{v}}_{b}∥/\alpha^{\left(k\right)}$ and the case-to-case matrix ${\tilde{T}}^{\left(k\right)}=∥{\tilde{s}}_{a}{\tilde{v}}_{b}∥/\left(\alpha^{\left(k\right)}\mu_{I}\right)$ of transmission per contact. Unfortunately, the counterparts for infection-to-infection, $R^{\left(k\right)}$ and $T^{\left(k\right)}$, are more biologically important. Because $s_{a}v_{a}=\left({\tilde{p}}_{a}s_{a}\right)\left(v_{a}/{\tilde{p}}_{a}\right)={\tilde{s}}_{a}{\tilde{v}}_{a}$, however, $R^{\left(k\right)}$ and $T^{\left(k\right)}$ have the same diagonal elements as ${\tilde{R}}^{\left(k\right)}$ and ${\tilde{T}}^{\left(k\right)}$. Even in the presence of subclinical infection, therefore, our statistical methods can always estimate the diagonal elements of $R^{\left(k\right)}$ and $T^{\left(k\right)}$, corresponding to epidemic spread within age-groups.

Like (Davies et al., 2020), we also consider three reduced models. We examine: (1) (constant clinical fraction) ${\tilde{p}}_{a}=\tilde{p}$; (2) (constant susceptibility) $s_{a}=s$; and (3) (constant infectivity) $v_{b}=v$. Our Constant Infectivity model $\left(v_{b}=v\right)$ is a natural reduced model for our full model, but not for the one in (Davies et al., 2020). For the Constant Clinical Fraction model $\left({\tilde{p}}_{a}=\tilde{p}\right),{\tilde{R}}^{\left(k\right)}=R^{\left(k\right)}$ and ${\tilde{T}}^{\left(k\right)}=T^{\left(k\right)}$; for the Constant Susceptibility model $\left(s_{a}=s\right),s_{a}v_{b}=s_{b}v_{b}={\tilde{s}}_{b}{\tilde{v}}_{b}$, so the rows of $R^{\left(k\right)}$ and $T^{\left(k\right)}$ are constant; and for Constant Infectivity model $\left(v_{b}=v\right),s_{a}v_{b}=s_{a}v_{a}={\tilde{s}}_{a}{\tilde{v}}_{a}$, so the columns of $R^{\left(k\right)}$ and $T^{\left(k\right)}$ are constant. For constant susceptibility or infectivity, therefore, the diagonals of ${\tilde{R}}^{\left(k\right)}$ and ${\tilde{T}}^{\left(k\right)}$ (which we can estimate) determine the full matrices $R^{\left(k\right)}$ and $T^{\left(k\right)}$. Thus, our methods determine $R^{\left(k\right)}$ and $T^{\left(k\right)}$ for all three reduced models.

## 3. Results

**Fig. 1.**
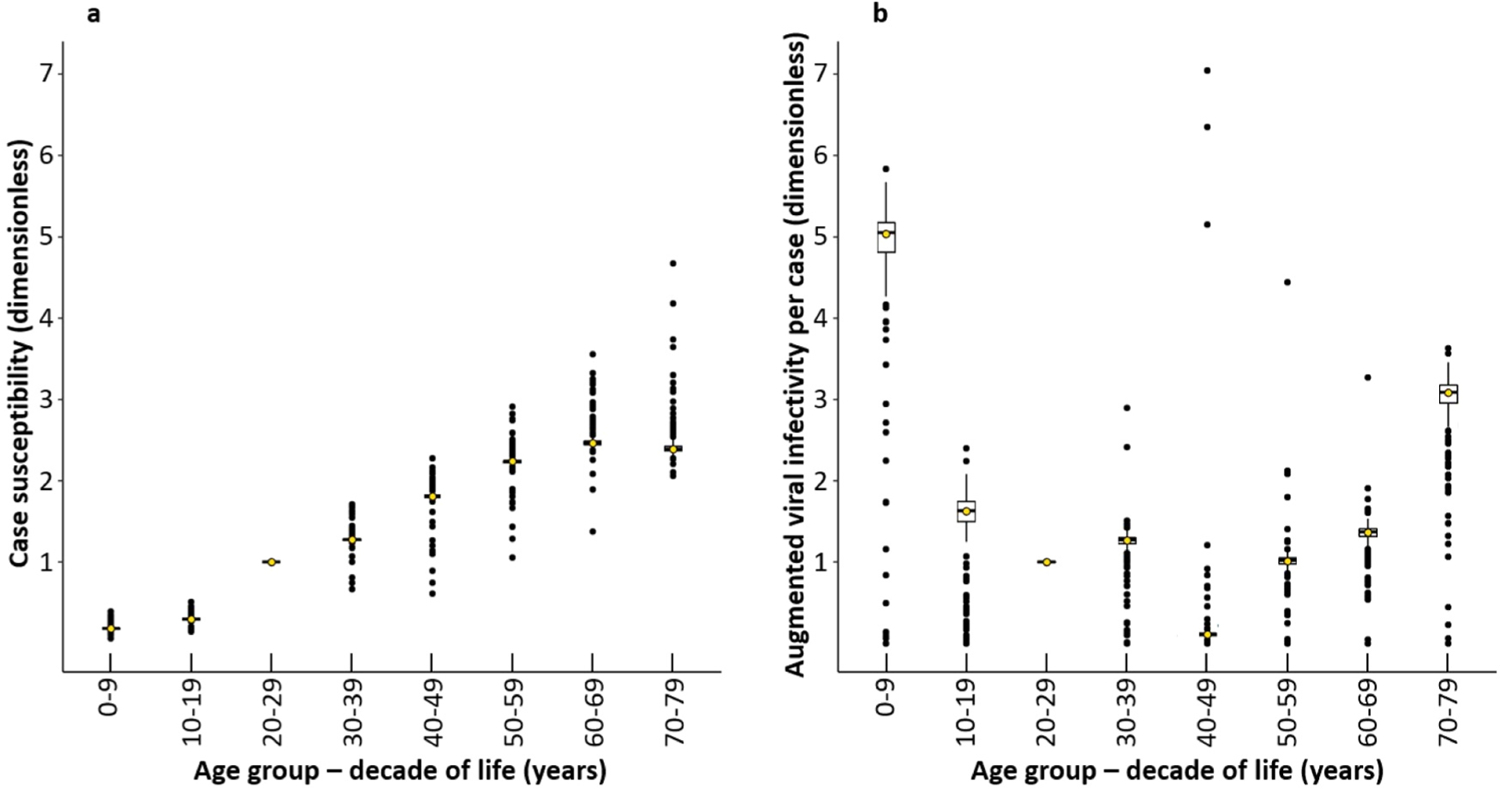
Box plots of susceptibility ${\tilde{s}}_{a}$ and augmented infectivity per case ${\tilde{v}}_{a}$ from 201 basin-hopping runs.

The fit in the Materials and Methods Section 2 used the regularized minimization of Eq. (7) to estimate ${\tilde{s}}_{a}$ and ${\tilde{v}}_{a}$ within each decade 0–9, 10–19, etc. In Fig. 1a on the left, the tick marks on the X-axis marks 8 age-groups, decades of life 0–9, 10–19, …, 70–79. The Y-axis represents the case susceptibility ${\tilde{s}}_{a}$. Fig. 1b on the right has the same X-axis, but the Y-axis represents the augmented (viral) infectivity per case ${\tilde{v}}_{a}$. In both graphs, for the mean $\chi^{2}=42$ and within each decade of life, the black points show the distinct local minima from 201 randomized runs of minimization. The computations normalized both ${\tilde{s}}_{a}$ and ${\tilde{v}}_{a}$ so the decade 20–29 yielded the value 1. Each of the boxplots within each decade 0–9, 10–19, etc., displays the median and two quartiles for the 201 local minima, often flattened to appear like a single horizontal line. The global minimum is represented by an open yellow circle, usually lying on the median line of the boxplot. Each of the points outside the interquartile boxes represents a distinct outlying local minimum from the 201 runs. On one hand, if an outlier is isolated, it could correspond to a distinctive subgroup within the decade of life. On the other hand, an outlier near other outliers likely represents a continuum of human variability or randomness in the data.

The Theory in Section 2.1 gives the biological interpretations for the quantities presented here. The case susceptibility in Stratum a (i.e., age-group a) is the product ${\tilde{s}}_{a}=s_{a}{\tilde{p}}_{a}$ of the susceptibility $s_{a}$ and the clinical fraction, the probability ${\tilde{p}}_{a}$ that an infection produces a case. The case susceptibility ${\tilde{s}}_{a}$ is therefore proportional to the probability that a single contact from typical infectious individual causes a case. The augmented (viral) infectivity per case is the quotient ${\tilde{v}}_{a}=v_{a}/{\tilde{p}}_{a}$ of the (viral) infectivity $v_{a}$ and ${\tilde{p}}_{a}$. The augmented infectivity per case ${\tilde{v}}_{a}$ is proportional to the expected number of infectious contacts from Stratum a per contact with a case in Stratum a, with each case surreptitiously augmented by infectious contacts from subclinical infections in Stratum a.

Fig. 1 shows vertical sets of points within bins for each decade of life: 0–9, 10–19, etc. Each set of points represent distinct local minima from 201 runs of regularized stochastic minimization, with the global minimum indicated by open circles (the first lines of the tabs “Standard (Black) “Regularized_Local_Minima.xlsx” of the Excel file in the SI displays the corresponding numerical values). Fig S1 shows that the regularized minimization was robust to the amount of smoothness imposed on the chi-square fit.

On one hand, for ${\tilde{s}}_{a}$ in Fig. 1a, the global minima increase almost linearly in decades after age 20 years. On the other hand, for ${\tilde{v}}_{a}$ in Fig. 1a, the global minima have a U-shaped pattern. Note: the factors ${\tilde{p}}_{a}$ in ${\tilde{s}}_{a}={\tilde{p}}_{a}s_{a}$ and ${\tilde{p}}_{a}^{-1}$ in ${\tilde{v}}_{a}=v_{a}/{\tilde{p}}_{a}$ obstruct direct conclusions about susceptibility $s_{a}$ and viral infectivity $v_{a}$. It would be fallacious to conclude summarily, e.g., that children and elderly are the most infectious strata, or that middle-aged people are the least infectious. Only some infections become cases, obstructing direct conclusions about $s_{a}$ and $v_{a}$.

At the resolution in Fig. 1, the boxes of median and quartiles of the local minima sometimes often appear to collapse into horizontal line segments. Usually, the global minimum is visually indistinguishable from the median in the boxes, however, indicating that the global minimum is representative of “typical” local minima. For ${\tilde{s}}_{a}$ in Fig. 1a, even outliers among the local minima are noticeably tight, suggesting the corresponding probability surfaces have funnels around the global minimum, fewer distinct local minima, and less heterogeneity within strata than for ${\tilde{v}}_{a}$ in Fig. 1b.

Fig. 1b displays estimates of ${\tilde{v}}_{a}=v_{a}/{\tilde{p}}_{a}$, showing that some local minima for ${\tilde{v}}_{a}$ are isolated outliers, far from the global minimum. For most decades of life, the outliers take smaller values of ${\tilde{v}}_{a}$ and are not particularly isolated, perhaps suggesting a continuum of human variability that includes reduced clinical fractions or viral infectivities. For the decades 40–49 and 50–59 of life, however, the outliers take larger values of ${\tilde{v}}_{a}$ and are isolated from each other. The isolated outliers could represent distinctive subgroups within the decade. If so, the subgroups may become less distinctive with aging as a cohort ages, eventually becoming confluent with the cohort, explaining the suppression of the corresponding local minima in later decades of life. In any case, the estimation for ${\tilde{v}}_{a}$ lacks the tightness and consistency of the local minima in the estimation of ${\tilde{s}}_{a}$.

For the six countries under present scrutiny, Table 1 gives the ISO 3166–1 alpha-3 country code, and the proportionality constant $\alpha^{\left(k\right)}$ yielding the case-to-case NGM in Eq. (5). The proportionality constant is 6.66±2.34 (sample average and standard deviation), a 2.34/6.66=35% relative error. The 35% relative error lies well within useful limits for public health decisions. The usefulness of the model justifies its approximations, e.g., that the Prem matrices can be taken without error, along with its other assumptions, e.g., that the six countries implicitly define their cases similarly.

In Table 1, the average α=6.66 is most closely approximated by $\alpha^{\left(k\right)}=6.11$ for Canada, with (6.66–6.11)/6.66 = 8.3% relative error. If required elsewhere, therefore, Canada can provide a country typical of our results.

**Fig. 2.**
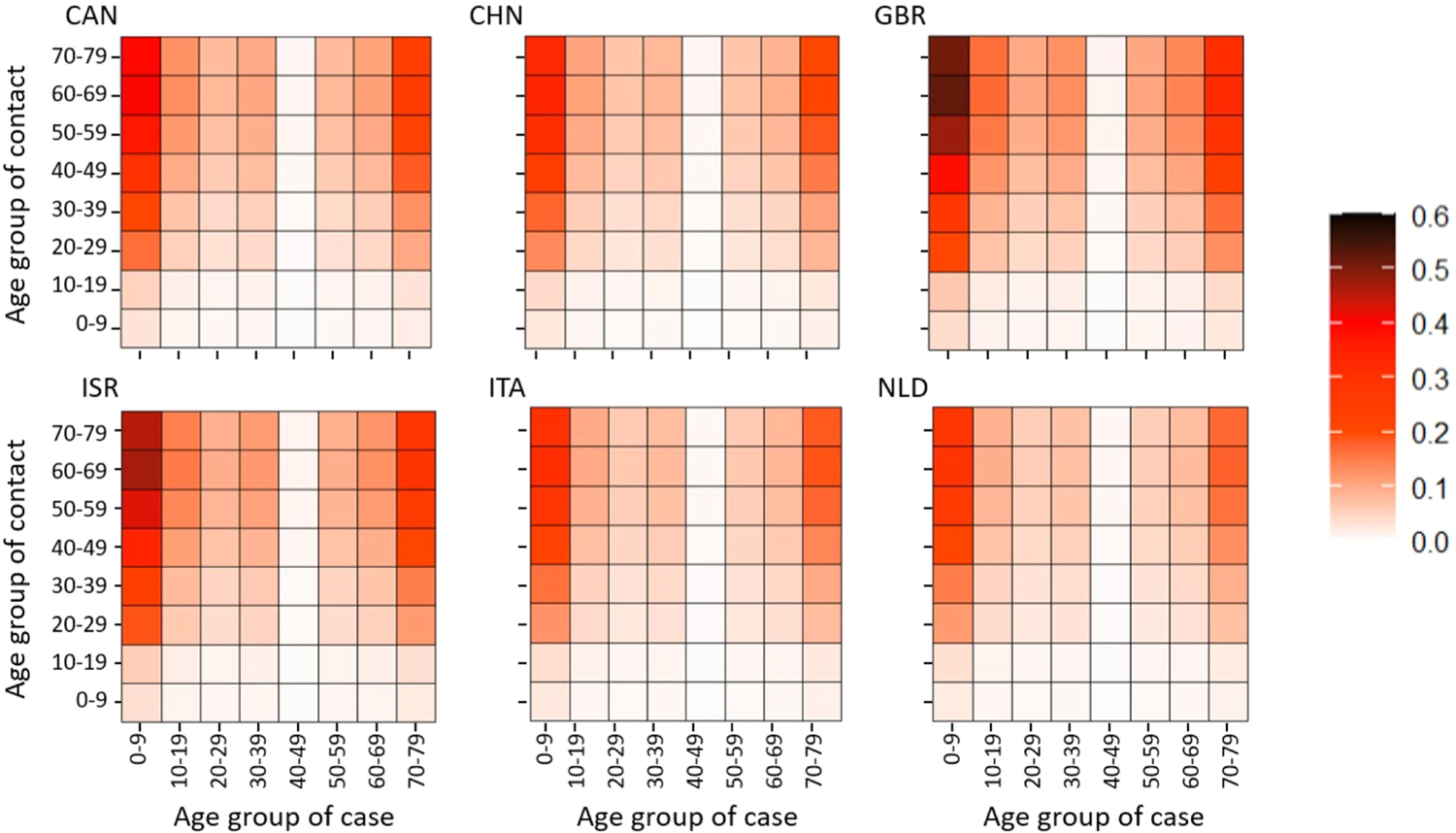
Heat maps representing the case-to-case transmission matrix for six countries.

Fig. 2 plots as heat maps in red the regularized estimates of the case-to-case matrix ${\tilde{T}}^{\left(k\right)}=\tilde{P}T^{\left(k\right)}{\tilde{P}}^{-1}$ of transmission per contact (defined in Section 2.2) for the six countries. The global minima in Fig. 1 provided the estimates of ${\tilde{s}}_{a}$ and ${\tilde{v}}_{a}$ used in computing $T^{\left(k\right)}$ for Fig. 2. Table 1 gives the ISO 3166–1 alpha-3 country code displayed in the upper left corner of each heat map to identify the country. Each heat map stratifies in decades, as in the case data. The X-axis gives the stratum of the case b and the Y-axis gives the stratum of the contact cases a infected in the next generation. The legend on the right of Fig. 2 gives the color values of the case-to-case transmission element ${\tilde{t}}_{a,b}^{\left(k\right)}=t_{a,b}^{\left(k\right)}\left(\tilde{p_{a}}/\tilde{p_{b}}\right)$. The matrix $T^{\left(k\right)}$ of transmission per contact and the case-to-case matrix ${\tilde{T}}^{\left(k\right)}=\tilde{P}T^{\left(k\right)}{\tilde{P}}^{-1}$ of transmission per contact therefore agree along the diagonal a=b, because ${\overset{ˆ}{t}}_{a,b}^{\left(k\right)}=t_{a,b}^{\left(k\right)}$ there.

Our equilibrium analysis determines some quantities important to early epidemics. Section 2.1 gives the biological interpretations for the matrix $T^{\left(k\right)}$ of transmission per contact in Eq. (2) and the case-to-case matrix ${\tilde{T}}^{\left(k\right)}$ of transmission per contact in Eq. (6). The dip in Fig. 1b for $\tilde{v}$ in the decade 40–49 causes a notable diminution in the corresponding column in ${\tilde{T}}^{\left(k\right)}$. As the text for Fig. 1 indicates, however, in the decade 40–49 the global minimum for $\tilde{v}$ may not be a good representative for all local minima, possibly because of distinctive outlying subgroups.

On one hand, as in Fig. 1, the factors ${\tilde{p}}_{a}$ in ${\tilde{s}}_{a}={\tilde{p}}_{a}s_{a}$ and ${\tilde{p}}_{a}^{-1}$ in ${\tilde{v}}_{a}=v_{a}/{\tilde{p}}_{a}$ obstruct direct conclusions off the diagonal a=b in Fig. 2. For example, Fig. 1 suggests that ${\tilde{p}}_{a}/{\tilde{p}}_{b}$ may be very large in the left upper corner of Fig. 2 inflating the case-to-case transmission from young to old. On the other hand, the diagonal elements of ${\tilde{T}}^{\left(k\right)}$ yield direct conclusions about the diagonal elements of $T^{\left(k\right)}$, because of equality. Perhaps most notably, transmission of infection per contact within decades 0–9 and 10–19 is small (less than about 0.1), much less than transmission per contact within decades 60–69 and 70–79 (about 0.1–0.4.).

**Fig. 3.**
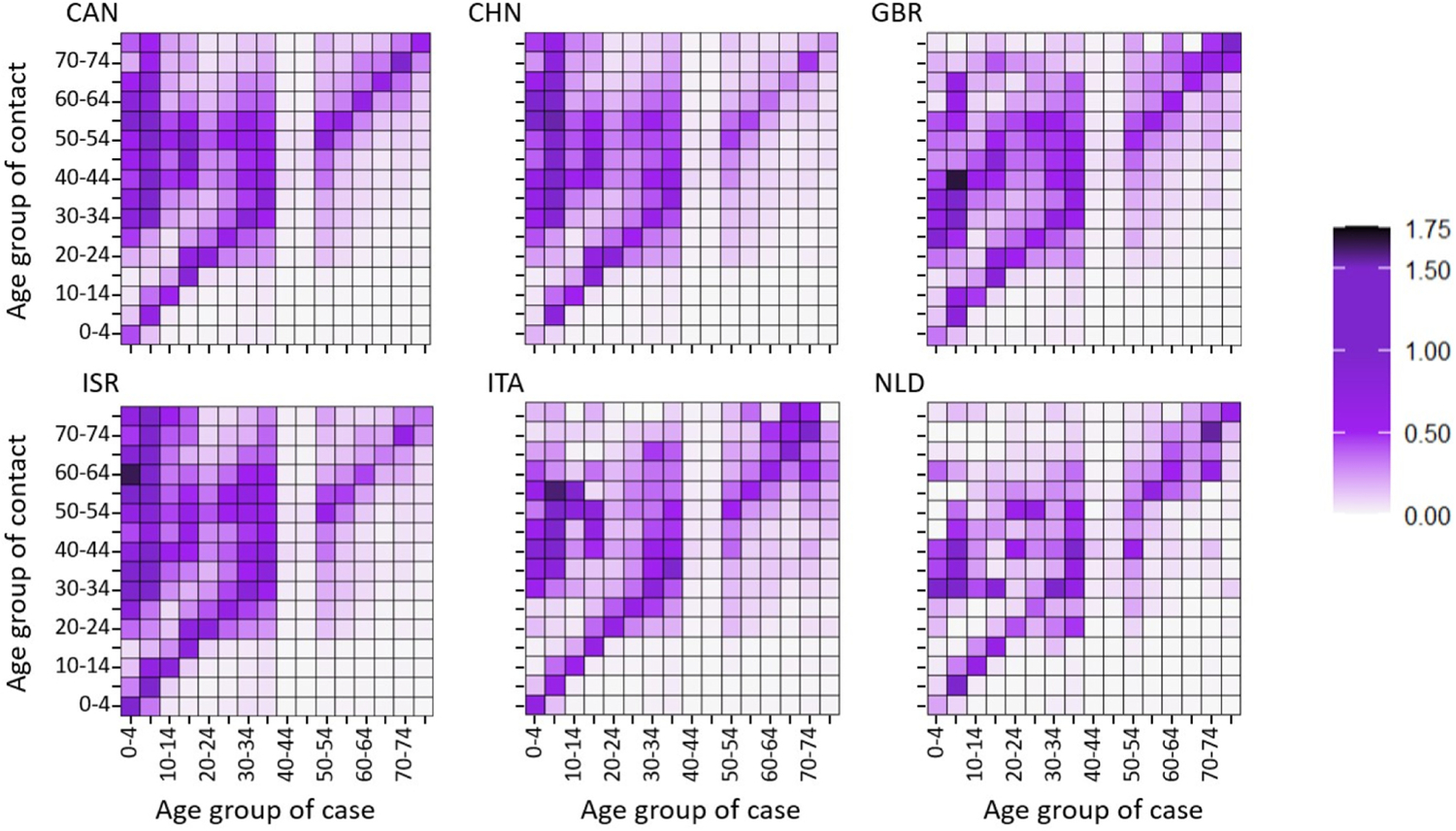
Heat maps representing the case-to-case $\text{NGM }{\tilde{\text{R}}}^{\left(k\right)}$ for six countries.

Fig. 3 plots as heat maps in purple for six countries the regularized estimates of the case-to-case NGM ${\tilde{\text{R}}}^{\left(k\right)}=\tilde{\text{P}}{\text{R}}^{\left(k\right)}{\tilde{\text{P}}}^{-1}$ (defined in Sections 2.1 and 2.2). The global minima in Fig. 1 provided the estimates of ${\tilde{s}}_{a}$ and ${\tilde{v}}_{a}$ used in computing $R^{\left(k\right)}$ for Fig. 3. (Prem et al., 2017) plot their contact matrices as blue heat maps. The purple heat maps for ${\tilde{\text{R}}}^{\left(k\right)}$ in Fig. 3 combine the Prem contact matrices with the red heat map for ${\tilde{\text{T}}}^{\left(k\right)}$ in Fig. 2. Fig. 3 uses the same layout as Fig. 2, but stratifies with the 16 half-decades of the Prem matrices. The X-axis gives the stratum of the case b and the Y-axis gives the stratum of the contact cases a infected in the next generation. The legend on the right of Fig. 3 gives the color values of the case-to-case reproduction number ${\tilde{r}}_{a,b}^{\left(k\right)}=r_{a,b}^{\left(k\right)}\left(\tilde{p_{a}}/\tilde{p_{b}}\right)$. The matrices $R^{\left(k\right)}$ and ${\tilde{\text{R}}}^{\left(k\right)}=\tilde{\text{P}}{\text{R}}^{\left(k\right)}{\tilde{\text{P}}}^{-1}$ therefore agree along the diagonal a=b, because ${\overset{˜}{r}}_{a,b}^{\left(k\right)}=r_{a,b}^{\left(k\right)}$ there.

Section 2.1 gives biological interpretations for the NGM $R^{\left(k\right)}$ in Eq. (3) and the case-to-case NGM ${\tilde{R}}^{\left(k\right)}$ in Eq. (5) and Fig. 3. Again, the dip in Fig. 1 for $\tilde{v}$ in the decade 40–49 causes a notable diminution in ${\tilde{R}}^{\left(k\right)}$ in the corresponding columns, and most of the caveats for ${\tilde{T}}^{\left(k\right)}$ in Fig. 2 propagate to ${\tilde{R}}^{\left(k\right)}$ in Fig. 3.

As in Fig. 2, the factors ${\tilde{p}}_{a}$ in ${\tilde{s}}_{a}={\tilde{p}}_{a}s_{a}$ and ${\tilde{p}}_{a}^{-1}$ in ${\tilde{v}}_{a}=v_{a}/{\tilde{p}}_{a}$ obstruct direct conclusions off the diagonal a=b in Fig. 3. Similarly, however, the diagonal elements of ${\tilde{R}}^{\left(k\right)}$ also yield direct conclusions about the diagonal elements of $R^{\left(k\right)}$, because of equality. Perhaps most notably, the rate of infectious contact within the half-decades from 5 to 40 generally shows no dramatic variation, and the half-decade 70–75 shows noticeably strong infectious contact on the diagonal in some countries.

## 4. Discussion

Many inferences relevant to public health policy during an epidemic can be made during its exponential phase. The present analysis extends a linear analysis of case data in the exponential phase during an influenza epidemic (Nishiura et al., 2010) to an infectious disease that can spread by asymptomatic infection, i.e., COVID-19. As anticipated, the linear analysis cannot replicate every conclusion revealed by a dynamic analysis using later case (Davies et al., 2020) or contact tracing data (Laxminarayan et al., 2020) (see, e.g., Section S2.1a). It can, however, draw its conclusions earlier and with far fewer disease-specific parameters, e.g., compare Table S1 (four items) to the Supplementary Table 1 in (Davies et al., 2020) (almost three times as many items). Our linear analysis used age-specific case data for each of six countries available publicly before April 23, 2020, so in principle our conclusions about COVID-19 were accessible shortly thereafter.

COVID-19 infection can be asymptomatic, so case counts have an intrinsic bias to underestimating the infection counts. To compensate, our model explicitly includes a clinical fraction varying with age, quantifying how often infection resulted in a recorded clinical case. As justified in Section 2.2, in each of six countries of interest, we drew our age-stratified case data from a single COVID-19 report, usually a two-weekly summary, dated near the end of the exponential phase.

By the six dates of the reports, less than 1.6% of the corresponding populations appear to have been tested (e.g., Canada, 2020; Netherlands, 2020; United_Kingdom, 2020). Thus, the corresponding clinical fractions contain biases that influence clinical attention. For example, the clinical fraction in the elderly may be biased upward because adult children might bring infection to clinical attention more readily for their parent than for themselves. Notably, however, our age-stratified case data is unlikely to suffer diurnal reporting biases, because the single report furnishing the data usually included cases over a two-week interval. In addition, operational definitions of a case are unlikely to change much in two weeks. Moreover, the relative constancy of $\alpha^{\left(k\right)}$ in Table 1 supports the assumption that the operational definitions of a case in the six countries of our study were comparable. Other sources of error, e.g., the varying sex ratios between countries (CIA, 2023), also fell within the 35% relative error of $\alpha^{\left(k\right)}$ in Table 1.

To understand our primary result in Fig. 3 better, consider (Laxminarayan et al., 2020), published in November of 2020. Laxminarayan et al. presented contact tracing data with testing specific for SARS-CoV-2 in the Indian states Uttar Pradesh and Tamil Nadu. Their Figures 2C9 and 2C10 display age-specific attack rates as matrices. Because the operational definition of the attack rates included diagnosis by test, in principle their attack matrices do not depend on clinical fractions. In fact, their attack matrices are closely related (but not identical) to age-specific next-generation matrices (NGMs), providing a useful contrast for the case-to-case NGMs in Fig. 3. The attack matrices are approximately diagonal, and Laxminarayan et al. infer from the diagonality that most of the COVID-19 transmission they examined took place within age-groups. In contrast, the elements in our case-to-case NGMs in Fig. 3 are close to 0 below the main diagonal but mostly positive elsewhere.

The following reconciles the contrast. The large superdiagonal elements in the case-to-case NGMs mostly reflect large ratios $\tilde{p_{a}}/\tilde{p_{b}}$ (contact over case) of the clinical fractions for different age-groups (see the equation ${\tilde{r}}_{a,b}^{\left(k\right)}=r_{a,b}^{\left(k\right)}\left(\tilde{p_{a}}/\tilde{p_{b}}\right)$ following Fig. 3). On one hand, a case among the young, particularly in half-decades less than 20 years, implies many subclinical infections. The subclinical infections effectively augment the effective force of infection from a case. On the other hand, a case among the elderly is unlikely to imply many other subclinical infections.

Contact tracing can in principle estimate the NGM. Moreover, a comparison of an NGM and our case-to-case NGM can estimate the age-specific clinical fraction at the beginning of an epidemic. Although such an estimation is beyond the purview of the present article, it could be useful after a diagnostic testing for infection has been developed but before its widespread availability. In any case, the major result of modeling in this article is that the diagonals of the NGM and the case-to-case NGM are the same. Importantly, the diagonal of the NGM corresponds to the within age-group transmission that dominated COVID-19 spread (Laxminarayan et al., 2020).

Our analysis omitted age-groups over 80, because the Prem contact matrices omitted the corresponding contact rates. Cases in age-groups above age 80 were about 3% in China and Israel; 7% for Canada; and 25% for the United Kingdom, Italy, and Netherlands). Fortunately, Fig. 1, Fig. 2, and Fig. 3 all establish clear trends by age-group.

Under the feasible assumption that the figures’ trends extend beyond age 80: (1) in Fig. 1, case susceptibility and augmented (viral) infectivity per case continue to increase beyond age 80; (2) the trends in Fig. 1 propagate to Fig. 2, which if extended, its values at northern and eastern borders of the case-to-case matrices of transmission per contact in Fig. 2 would continue to increase beyond age 80; and (3) in Fig. 3, which if similarly extended, the subdiagonal values in the scaled next-generation matrices would remain small, a social property inherited from the Prem contact matrices. In other words, the age-groups over age 80 omitted in the data fit are likely to have both high case susceptibility and high augmented infectivity per case, but their limited social contacts with younger age-groups would likely have curtailed COVID-19 spread from them to younger age-groups. It is therefore improbable that the omission of the over-80 age-group would have changed our scientific conclusions about COVID-19 spread among the younger age-groups.

(Hortaçsu et al., 2021) contains many references to estimates of the clinical fraction in COVID-19. When estimating the basic reproduction number $R_{0}$ during exponential growth (Zhao et al., 2020), “it is often difficult to identify [the clinical fraction] alongside the growth of the infection purely by measures of fit” (Hortaçsu et al., 2021). Eqs. (3) and (4) (see also Section S2.1) show that if the spread from subclinical infections and cases is similar, then once the population distribution has equilibrated, subclinical infections and cases grow in parallel at the same rate, i.e., their basic reproduction number $R_{0}$ and their growth rate r are the same (see Section S2.1). The parallel growth suggests that in practice, after the infected population has equilibrated, the clinical fraction may be impossible to identify without a diagnostic test for subclinical infections.

Results unique to dynamic analysis depend on transients, not equilibria, however. On one hand, before the equilibration of the infected population during the exponential phase, infections are usually relatively sparse and correspondingly uninformative. On the other hand, after the infected population has equilibrated, with NPIs and other transients perturbing the equilibrium, biological interpretation becomes more complex. Moreover, dynamic analyses requires initial conditions, and model assumptions about epidemic seeding can appear somewhat arbitrary (e.g., Davies et al., 2020).

Dynamic analysis yields more detailed results than linear analysis, however. The present linear analysis identifies only the product of the age-specific susceptibility and clinic fraction, e.g., not the two parameters separately (see the left of Fig. 1). Perhaps somewhat surprisingly in view of the observations from (Hortaçsu et al., 2021) above, a dynamic analysis can apparently identify the age-specific susceptibility and clinic fraction separately from case data alone (Davies et al., 2020). To distinguish the stages of infection in individual cases (Harris et al., 2023), the separate identification must depend on transients in case counts (possibly heightened by NPIs), with all the logical dependencies the a dynamic model entails. If the full model is reduced by setting either susceptibility and clinic fraction to a constant (e.g., if infections were always detectible), however, the linear analysis can then estimate the disease parameters unambiguously (see comments relevant to Fig. 2 and Fig. 3 just before the Results).

In any case, public health decisions during the COVID-19 pandemic demonstrated that knowledge has much greater impact when gained early in an epidemic, motivating us to compare our results from the exponential phase with conclusions later in the epidemic. The left of Fig. 1 shows that that the product of susceptibility and the tendency to display infection increase with age, as found in (Davies et al., 2020) (cf. also Dattner et al., 2021). In fact, the shape in Fig. 1 agrees qualitatively with plots for susceptibility and clinical fraction in Figs. 1b and 1c of (Davies et al., 2020), who noted the similarity of their Figs. 1b and 1c.

Fig. 1 hints subtly but objectively that some distinctive subgroups within the decades 40–49 and 50–59 have anomalously high infectivity. Unfortunately, we could not find specific data to corroborate the existence of such subgroups. One study on Omicron infection in the elderly (Wang et al., 2023) associated inflammation and Ct values for upper respiratory viral RNA. Although remaining speculative, therefore, the distinctive subgroups in Fig. 1 may correspond to specific constellations of immune derangements (e.g., autoimmune diseases) that lose distinctiveness as the 50–59 year-old cohort ages.

As mentioned in the Results, the diagonals of Fig. 2 estimate directly the average transmissions per contact within each age-group. The values on the diagonals of Fig. 2 rise steadily with age, except for an anomalous dip within the decade 40–49, approximately anticipating the same phenomenon in Figs C9 and C10 for the attack rates from testing in (Laxminarayan et al., 2020). The diagonals in Fig. 2 and Fig. 3 generally support conclusions elsewhere (Davies et al., 2020) that school closures during the start of the COVID-19 pandemic were unlikely to impact COVID-19 as much as they impacted influenza epidemics (cf. also Dattner et al., 2021; Monod et al., 2021).

Fig. 3 combines the transmission matrix with the Prem contact matrices to draw conclusions about epidemic spread in different countries. The diagonals of Fig. 3 present the elements of the age-specific NGM, which govern spread within the age-groups. They show that with the inconsistent exception of the half-decade 0–4 and the anomaly of the decade 40–49 from the transmission matrix, the reproduction numbers within the younger half-decades (e.g., 5–40) tend to be larger than older half-decades, with the notable exception of the half-decade 70–74 in some countries (cf. Irfan et al., 2021; Lau et al., 2020; Monod et al., 2021; Rostad et al., 2021). Old-age homes became hot-spots for epidemic mortality (Daly et al., 2022). Retrospectively, although the homes were known to display epidemic seeding from sharing employees and importing infected residents, Fig. 3 actionably points to the younger residents as a danger for spreading infection, presumably because of their higher sociability (Mizumoto et al., 2020; Russell et al., 2020).

The form of our model for the age-dependence of susceptibility, infectivity, and subclinical infection deliberately mirrors a ground state for epidemiological ignorance before more complex models can incorporate specific information about subclinical infection. (Nishiura et al., 2010) provided most of the mathematical framework of our ground state model, with two important exceptions. Where they modeled contact $c_{a,b}$ and susceptibility $s_{a}$ (a property of the individual a receiving contact), we added infectivity $v_{b}$ (a property of the individual b giving contact) and the clinical fraction, the probability that an infection becomes a case $\left({\tilde{p}}_{a}\right)$. Theoretical estimates of infectivity complement its practical quantitation, which can be difficult (Han et al., 2021). The role of age-specific infectivity per contact in our analysis accorded with the practical observation that infectivity is an important variable in respiratory illness (see the right of Fig. 1), e.g., children may be much less likely to sneeze into their elbows than adults. Our primary aim here was not to provide a detailed statistical retrospective on COVID-19, however, but to explore further possibilities for early epidemic analysis.

## Supplementary Material

MMC2

MMC1

## Figures and Tables

**Table 1:** Each country k with its proportionality constant $\alpha^{\left(k\right)}$ from the fit.

Code	Country *k*	*a* ^(*k*)^
CAN	Canada	6.11
CHN	China	7.36
GBR	United Kingdom	4.74
ISR	Israel	5.29
ITA	Italy	7.98
NLD	Netherlands	8.61

## Data Availability

All data and code are publicly available. https://github.com/Zakstanke/CovidStratified. The data, code, and scripts for the analysis and regularized chi-square estimation are available at GitHub.
