# Supplementary material for: Estimating age-stratified transmission and reproduction numbers during the early exponential phase of an epidemic: A case study with COVID-19 data
Source: Epidemics. Author manuscript; Available in PMC 2023 Sep 27. (PMC10528737; doi:10.1016/j.epidem.2023.100714)
Supplement: MMC1 [file NIHMS1927259-supplement-MMC1.docx]

# Supplementary Information (SI)

The SI fills in details for the main article, which should be read first.

Sections and tables within this supplementary text are preceded by “S”, e.g., Section S1 and Table S1. Rather than explicitly and repeatedly referring the reader of the main text to the SI for mathematical details, the sections within the SI correspond to sections in the main text, and except for the prefix “S” are numbered similarly. Some sections within the SI have a postfix “a”, e.g., S2.1a, to indicate that they are ancillary to a section in the main text, but without direct correspondence to the material there. Below, sections, figures, and tables without “S” refer to the main article.

# Methods and Materials

## S2.1 Theory

Let denote mathematical expectation; “$:=$”, a definition. Following standard theory ([Wallinga and Lipsitch, 2007](#_ENREF_19)), consider any infectious disease with random generation time $T$ (e.g., COVID-19) and moment generating function

.

Reflecting ignorance in an early epidemic, we assume that the distribution of $T$ is the same for all infections, regardless of age-group or country ([Davies et al., 2020](#_ENREF_3)), so Eq is valid for every infection (see a similar analysis of influenza ([Nishiura et al., 2010](#_ENREF_12))).

Given a square matrix $\mathbf{A}$, the spectral radius $\rho\left(\mathbf{A}\right)$ denotes the largest absolute value of its eigenvalues. In general, the basic reproduction number $R_{0}=\rho\left(\mathbf{R}\right)$, where $\mathbf{R}$ is the NGM (([Diekmann et al., 1990](#_ENREF_5)) and e.g., ([Diekmann et al., 2010](#_ENREF_6); [van den Driessche and Watmough, 2002](#_ENREF_18); [Wallinga and Lipsitch, 2007](#_ENREF_19))). The spectral radius satisfies both $\rho\left(a\mathbf{R}\right)=a\rho\left(\mathbf{R}\right)$ for $a>0$ and $\rho\left(\mathbf{R}\right)=\rho\left(\tilde{\mathbf{P}}\mathbf{R}\tilde{\mathbf{P}}^{-1}\right)=\rho\left(\tilde{\mathbf{R}}\right)$ ([Lancaster and Tismenetsky, 1985, Ex 4.14.10b](#_ENREF_11)).

Let $r$ denote the initial exponential growth rate of the epidemic in a country. The theory of Markov renewals in probability theory ([Asmussen, 2003](#_ENREF_1)) or of linear operators in functional analysis (e.g., Section 8.2 of ([Diekmann et al., 2013](#_ENREF_4))) shows that in the present context, the Euler Lotka equation (2.4) of ([Wallinga and Lipsitch, 2007](#_ENREF_19)) generalizes here to the equation $G\left(-r\right)\mathbf{Ru}=\mathbf{u}$, where $\mathbf{u}$ is the non-negative dominant (Perron-Frobenius) eigenvector of the non-negative matrix $\mathbf{R}$ ([Seneta, 1981](#_ENREF_16)). The factored form $G\left(-r\right)\mathbf{R}$ requires the assumption that subclinical infections and cases spread infection similarly (e.g., ([Nishiura et al., 2010](#_ENREF_12))) and may not pertain without it. Thus, the Perron-Frobenius theorem ([Seneta, 1981](#_ENREF_16)) yields

$\rho\left(G\left(-r\right)\mathbf{R}\right)=G\left(-r\right)\rho\left(\mathbf{R}\right)=G\left(-r\right)\rho\left(\tilde{\mathbf{R}}\right)=1$.

Because of Eq (3), $G\left(-r\right)R_{0}=1$. With age-groups aggregated, therefore, both cases and infection both have the same overall basic reproduction number $\rho\left(\mathbf{R}\right)=\rho\left(\tilde{\mathbf{R}}\right)=R_{0}$ (see Eqs (3) and (4)), so cases and infection both have the same exponential growth rate $r$.

## S2.1a A Special Case of Eq (3) Relevant to ([Davies et al., 2020](#_ENREF_3))

Eqs (1) and (5) can include models that average the effects of subclinical infection. Under a model where infections evolve randomly and independently into subclinical infections and cases, and for this subsection only, define $\underline{\mathbf{v}}:=\left[\underline{v}_{a}\right]$ and $\bar{\mathbf{v}}:=\left[\bar{v}_{a}\right]$, column vectors separately characterizing the infectivity $v_{a}$ for subclinical infections and cases. An infection in Stratum $a$ therefore has average infectivity $v_{a}=\underline{v}_{a}\left(1-\tilde{p}_{a}\right)+\bar{v}_{a}\tilde{p}_{a}$. After normalizing $\underline{\mathbf{v}}$ and $\bar{\mathbf{v}}$, in a model where strata have equal infectivity (as in ([Davies et al., 2020](#_ENREF_3))), let $\bar{v}_{a}=1$ for a case and $\underline{v}_{a}=f$ for an clinical infection, yielding $v_{a}=f\left(1-\tilde{p}_{a}\right)+\tilde{p}_{a}$. The definition $\tilde{\mathbf{v}}=\tilde{\mathbf{P}}^{-1}\mathbf{v}$ following Eq (4) yields (within a constant of proportionality)

$\tilde{\mathbf{V}}=\text{diag}\left(\tilde{\mathbf{v}}\right)=\tilde{\mathbf{P}}^{-1}\left[f\left(\mathbf{I}-\tilde{\mathbf{P}}\right)+\tilde{\mathbf{P}}\right]=f\left(\tilde{\mathbf{P}}^{-1}-\mathbf{I}\right)+\mathbf{I}$,

a special case similar to the model in ([Davies et al., 2020](#_ENREF_3)).

Averages can therefore break the symmetry between subclinical infections and cases, so our models can handle more than just the ground state model of epidemiological ignorance.

## S2.2 Data Fit

The following parameter values were derived elsewhere.

**Table S1. Disease parameters for COVID-19 used in the present article**.

| **Parameter** | **Value** | **Description** | | **Source** |
| --- | --- | --- | --- | --- |
| $\mu$ | 5.40 days | | generation time mean | ([Rai et al., 2021](#_ENREF_15)) |
| $\sigma$ | 1.72 days | | generation time standard deviation | ([Ganyani et al., 2020](#_ENREF_8)) |
| $\kappa$ | 0.101 | | generation time dispersion | ([Spouge, 2021](#_ENREF_17)) |
| $\tau_{I}$ | 5 days | | infectious period | ([Davies et al., 2020](#_ENREF_3)) |

In early 2020, the state of knowledge about the parameters of COVID-19 was limited. The random generation time $T$ in was typically assumed to follow a $\text{Gamma}\left(\mu,\kappa\right)$ distribution (see Table S1). ([Spouge, 2021](#_ENREF_17)) took the distribution’s mean as $\mu$=5.40 ([Rai et al., 2021](#_ENREF_15)) and its standard deviation as $\sigma$=1.72 ([Ganyani et al., 2020](#_ENREF_8)), yielding the dispersion factor $\kappa=\sigma^{2}/\mu^{2}$=0.101. Table S1 therefore uses parameters that post-date early 2020, but it permits backward comparison with ([Spouge, 2021](#_ENREF_17)).

([Harris et al., 2023](#_ENREF_9)) note that symptomatic and asymptomatic transmission have different time scales. In early 2020, however, Davies et al ([Davies et al., 2020](#_ENREF_3)) estimated the average duration of preclinical infectivity as 2.1 days from ([Backer et al., 2020](#_ENREF_2)); of clinical infectivity as 2.9 days from ([Kucharski et al., 2020](#_ENREF_10)). These estimates yield a total mean time $\tau_{I}$ of infectivity as $\tau_{I}$=5.0=2.1+2.9 days ([Davies et al., 2020](#_ENREF_3)). They used the same estimate for the average duration of subclinical infectivity, so we do the same here.

**Table S2. Each country *k* with its basic reproduction number** $R_{0}^{\left(k\right)}$ **from (**[**Spouge, 2021**](#_ENREF_17)**)**.

| **Code** | **Country *k*** | ***R*_0_(*^k^*)** |
| --- | --- | --- |
| CAN | Canada | 2.74 |
| CHN | China | 2.23 |
| GBR | United Kingdom | 2.58 |
| ISR | Israel | 2.94 |
| ITA | Italy | 2.82 |
| NLD | Netherlands | 2.23 |

The exponential method ([Wallinga and Lipsitch, 2007](#_ENREF_19)) provided $R_{0}^{\left(k\right)}$ from case data for the Country $k$ ($k=1,...,K$).Table S2 displays the ISO 3166-1 alpha-3 country code and the basic reproduction number $R_{0}^{\left(k\right)}$ from ([Spouge, 2021](#_ENREF_17)). ([Prem et al., 2017](#_ENREF_13)) give the corresponding six Prem matrices $\mathbf{C}^{\left(k\right)}$.

See Sections 2.2 and 2.3 for notation. Until indicated otherwise, the discourse is restricted to any single Country $k$ and a health report from any time-interval late in its exponential phase. A random case falls into the $i$-th decade of life ($i=1,...,m$) with empirical probability $\hat{u}_{i,\bullet}^{\left(k\right)}:=\tilde{n}_{i,\bullet}^{\left(k\right)}/\tilde{n}_{\bullet,\bullet}^{\left(k\right)}$, approximately equaling the underlying equilibrium probability $\tilde{u}_{i,\bullet}^{\left(k\right)}=\tilde{u}_{i,\bullet}^{\left(k\right)}\left(\mathbf{\theta}\right)$ of Section 2.3.

Many countries stratified COVID-19 case data by decade, whereas the Prem contact matrices stratified by half decades up to age 80. As addressed in the Discussion Section 4, we omitted data for over-80 age-groups from our analysis and used only the stratified case data for the first $m$=8 decades of life. Our computations retained matrices of 16 dimensions, however, to leverage the full half-decade resolution of the Prem matrix.

Assume that the disease parameters $\mathbf{\theta}=\left(\tilde{\mathbf{s}},\tilde{\mathbf{v}}\right)$ apply to all countries, with the half-decade case counts being independent Poisson variates whose means are functions of the 14 unknown model parameters $\mathbf{\theta}=\left(\tilde{\mathbf{s}},\tilde{\mathbf{v}}\right)$ given by Fig 1. Conditioned on the total cases $\tilde{n}_{\bullet,\bullet}^{\left(k\right)}$ in Country $k$, the distribution of $\left\|\tilde{n}_{i,\bullet}^{\left(k\right)}\right\|$ then follows a multinomial distribution with probabilities $\tilde{\mathbf{u}}^{\left(k\right)}=\left\|\tilde{u}_{i,\bullet}^{\left(k\right)}\right\|=\left\|\tilde{u}_{2i-1}^{\left(k\right)}+\tilde{u}_{2i}^{\left(k\right)}\right\|$, where Eqs (4) and (5) determine the eigenvector $\tilde{\mathbf{u}}^{\left(k\right)}$ of dimension $2m=16$ as an function of $\mathbf{C}^{\left(k\right)}$ and $\mathbf{\theta}=\left(\tilde{\mathbf{s}},\tilde{\mathbf{v}}\right)$.

## S2.2a The Maximum Likelihood Estimation

The rest of the Subsection 2.2 describes the direct maximum likelihood estimation. Because it failed, only readers skeptical of the need for regularization need read it.

The analysis now considers case data $\mathbf{D}:=\left[\mathbf{D}^{\left(k\right)}\right]$ for all countries, where $\mathbf{D}^{\left(k\right)}:=\left[\tilde{n}_{i,\bullet}^{\left(k\right)}\right]$ gives the case counts in Country $k$. Assume that the countries are probabilistically independent. Eq (5) determines the eigenvector $\left[\tilde{u}_{a}^{\left(k\right)}\left(\mathbf{\theta}\right)\right]$ of dimension 16 as a function of $\mathbf{C}^{\left(k\right)}$, $R_{0}^{\left(k\right)}$, and the current parameter estimates $\mathbf{\theta}=\left(\tilde{\mathbf{s}},\tilde{\mathbf{v}}\right)$.

The negative log-likelihood satisfies

$\begin{matrix} -\ln p\left(\mathbf{D}\left|\mathbf{\theta}\right.\right)=-\sum_{\left(k\right)} \ln p\left(\mathbf{D}^{\left(k\right)}\left|\mathbf{\theta}\right.\right) \\ \equiv-\sum_{\left(k\right)} \sum_{i=1}^{m} \tilde{n}_{i,\bullet}^{\left(k\right)}\ln\tilde{u}_{i,\bullet}^{\left(k\right)}\left(\mathbf{\theta}\right) \\ \equiv-\sum_{\left(k\right)} \sum_{i=1}^{m} \tilde{n}_{i,\bullet}^{\left(k\right)}\ln\tilde{u}_{i,\bullet}^{\left(k\right)}\left(\mathbf{\theta}\right)+\sum_{\left(k\right)} \sum_{i=1}^{m} \tilde{n}_{i,\bullet}^{\left(k\right)}\ln\hat{u}_{i,\bullet}^{\left(k\right)} \\ =\sum_{\left(k\right)} \tilde{n}_{\bullet,\bullet}^{\left(k\right)}\sum_{i=1}^{m} \hat{u}_{i,\bullet}^{\left(k\right)}\ln\frac{\hat{u}_{i,\bullet}^{\left(k\right)}}{\tilde{u}_{i,\bullet}^{\left(k\right)}\left(\mathbf{\theta}\right)} \end{matrix}$,

with the following explanation. The first equality follows from the assumed independence of countries $k$. The ‘’ on the second line indicates that the right-hand expressions in the first and second lines differ by a number depending on only data. The difference is therefore irrelevant to the minimization determining the parameter $\mathbf{\theta}$. In this case, the difference is the logarithm of the multinomial coefficient in the multinomial probability $p\left(\mathbf{D}^{\left(k\right)}\left|\mathbf{\theta}\right.\right)$. The ‘’ on the third line indicates the addition of the second term. The second term depends on only data and is also therefore irrelevant to the minimization. The equality on the fourth line reflects the equality $\hat{u}_{i,\bullet}^{\left(k\right)}=\tilde{n}_{i,\bullet}^{\left(k\right)}/\tilde{n}_{\bullet,\bullet}^{\left(k\right)}$.

We used randomized basin-hopping from the Python optimize library to estimate $\mathbf{\theta}=\left(\tilde{\mathbf{s}},\tilde{\mathbf{v}}\right)$ by maximum likelihood. Differences between randomized runs produced wildly different estimates, each estimate usually having a different single coordinate of $\mathbf{\theta}$ much larger than others. The differing estimates suggested an ill-conditioned problem ([Press et al., 1976, p.61](#_ENREF_14)). Another attempt at maximum likelihood estimation using a package for profile likelihood ([Fischer and Lewis, 2021](#_ENREF_7)) warned explicitly about ill-conditioned estimates. The ill-conditioning suggested estimating $\mathbf{\theta}$ with the regularization ([Press et al., 1976, p.808](#_ENREF_14)) in Section S2.3.

## S2.3 **Data Fit with Regularization**

The Tikhonov-Miller regularization ([Press et al., 1976, p.808](#_ENREF_14)) provides a standard antidote to ill-conditioning by insisting on a smooth solution, but without insisting on a specific functional form, e.g., a half-cosine ([Davies et al., 2020](#_ENREF_3)). We minimized a regularized expression $F\left(\lambda,\mathbf{\theta}\right)=A\left(\mathbf{\theta}\right)+\lambda B\left(\mathbf{\theta}\right)$, where the chi-square term $A\left(\mathbf{\theta}\right)=\chi^{2}\left(\hat{\mathbf{u}}\left\|\mathbf{\theta}\right.\right)$ from Eq (7) has $N=42$ degrees of freedom. To express prior belief about smoothness, let

$$B\left(\mathbf{\theta}\right):=\sum_{i=1}^{m-2} \left(\tilde{s}_{2i}-2\tilde{s}_{2\left(i+1\right)}+\tilde{s}_{2\left(i+2\right)}\right)^{2}+\sum_{i=1}^{m-2} \left(\tilde{v}_{2i}-2\tilde{v}_{2\left(i+1\right)}+\tilde{v}_{2\left(i+2\right)}\right)^{2}$$

for $\mathbf{\theta}=\left(\tilde{\mathbf{s}},\tilde{\mathbf{v}}\right)\tilde{\mathbf{v}}$. The quadratic form of $B\left(\mathbf{\theta}\right)$ implicitly corresponds to Bayesian regularization using a prior Gaussian distribution. The subscripts’ factors of 2 reflect the half-decade constraints $\tilde{s}_{2i-1}=\tilde{s}_{2i}$ and $\tilde{v}_{2i-1}=\tilde{v}_{2i}$ that make $\left[s_{a}\right]$ and $\left[v_{a}\right]$ constant over the $i$-th decade of life ($i=1,2,...,m$). Thus, if $\tilde{\mathbf{s}}$ (or $\tilde{\mathbf{v}}$) changes linearly with each successive decade, there is no contribution to $B\left(\mathbf{\theta}\right)$. The normalizations $\tilde{s}_{5}=\tilde{s}_{6}=1$ and $\tilde{v}_{5}=\tilde{v}_{6}=1$ in Section 2.2. make $\tilde{s}_{a}$ and comparable in size, so Eq weights their smoothness similarly. For fixed $\lambda$, the regularization ([Press et al., 1976, p.808](#_ENREF_49)) minimizes $F\left(\lambda,\mathbf{\theta}\right)=A\left(\mathbf{\theta}\right)+\lambda B\left(\mathbf{\theta}\right)$, where $\lambda$ implicitly weights prior belief in smoothness against the closeness of the data fit. Our regularization implemented the basin-hopping minimization mentioned above. Statistical theory optimizes $\lambda$ in a principled manner as follows.

The expression $\chi^{2}\left(\hat{\mathbf{u}}\left\|\mathbf{\theta}\right.\right)$ in Eq (7) has 42 degrees of freedom, making its mean 42; its variance, 84=2*42. Randomness yields $\chi^{2}\left(\hat{\mathbf{u}}\left\|\mathbf{\theta}\right.\right)$=42 on average, with random fluctuations typically putting $\chi^{2}\left(\hat{\mathbf{u}}\left\|\mathbf{\theta}\right.\right)$ in the interval [42-√84, 42+√84]. Let the minimization estimate values $\lambda=\lambda_{-},\lambda_{0},\lambda_{+}$ leading to $\chi^{2}\left(\hat{\mathbf{u}}\left\|\mathbf{\theta}\right.\right)$=42-√84, 42, and 42+√84. The corresponding minimizing values of $\mathbf{\theta}_{\mathbf{-}},\mathbf{\theta}_{\mathbf{0}},\mathbf{\theta}_{+}$ indicate the size of random fluctuations in regularized parameter estimates, indicating the sensitivity of regularized parameter estimates to smoothing.

The normalizations $\tilde{s}_{5}=\tilde{s}_{6}=1$ and $\tilde{v}_{5}=\tilde{v}_{6}=1$ impose a magnitude of order 1 the 12 terms in Eq , so we initialized the binary search with $\lambda=\lambda^{\left(0\right)}=N/\left[2\left(m-2\right)\right]=42/12$, initially giving the data fit term $\chi^{2}\left(\hat{\mathbf{u}}\left\|\mathbf{\theta}\right.\right)$ and the smoothness term $B\left(\mathbf{\theta}\right)$ comparable influence on the iterative estimates of $\lambda=\lambda_{-},\lambda_{0},\lambda_{+}$ and the regularized fit.

## S2.3a The Chi-square Approximation of Maximum Likelihood Estimation

To justify replacing the minus log-likelihood in Eq with the chi-square approximation $\chi^{2}\left(\hat{\mathbf{u}}\left\|\mathbf{\theta}\right.\right)$ in Eq (7), consider any probability distribution $\mathbf{q}$ “close to” another probability distribution $\mathbf{p}$. Then, by noting $\sum_{i=1}^{m} p_{i}=\sum_{i=1}^{m} q_{i}=1$,

$\begin{matrix} \sum_{i=1}^{m} q_{i}\ln\frac{q_{i}}{p_{i}}=-\sum_{i=1}^{m} q_{i}\ln\left(1-\frac{q_{i}-p_{i}}{q_{i}}\right) \\ \approx\sum_{i=1}^{m} q_{i}\left[\left(\frac{q_{i}-p_{i}}{q_{i}}\right)+\frac{1}{2}\left(\frac{q_{i}-p_{i}}{q_{i}}\right)^{2}\right] \\ \approx\frac{1}{2}\sum_{i=1}^{m} \frac{\left(q_{i}-p_{i}\right)^{2}}{q_{i}} \end{matrix}$.

Substitute $q_{i}=\hat{u}_{i,\bullet}^{\left(k\right)}$ and $p_{i}=\tilde{u}_{i,\bullet}^{\left(k\right)}\left(\mathbf{\theta}\right)$ into Eq to yield Eq (7) (with an irrelevant factor of ½).

# Results


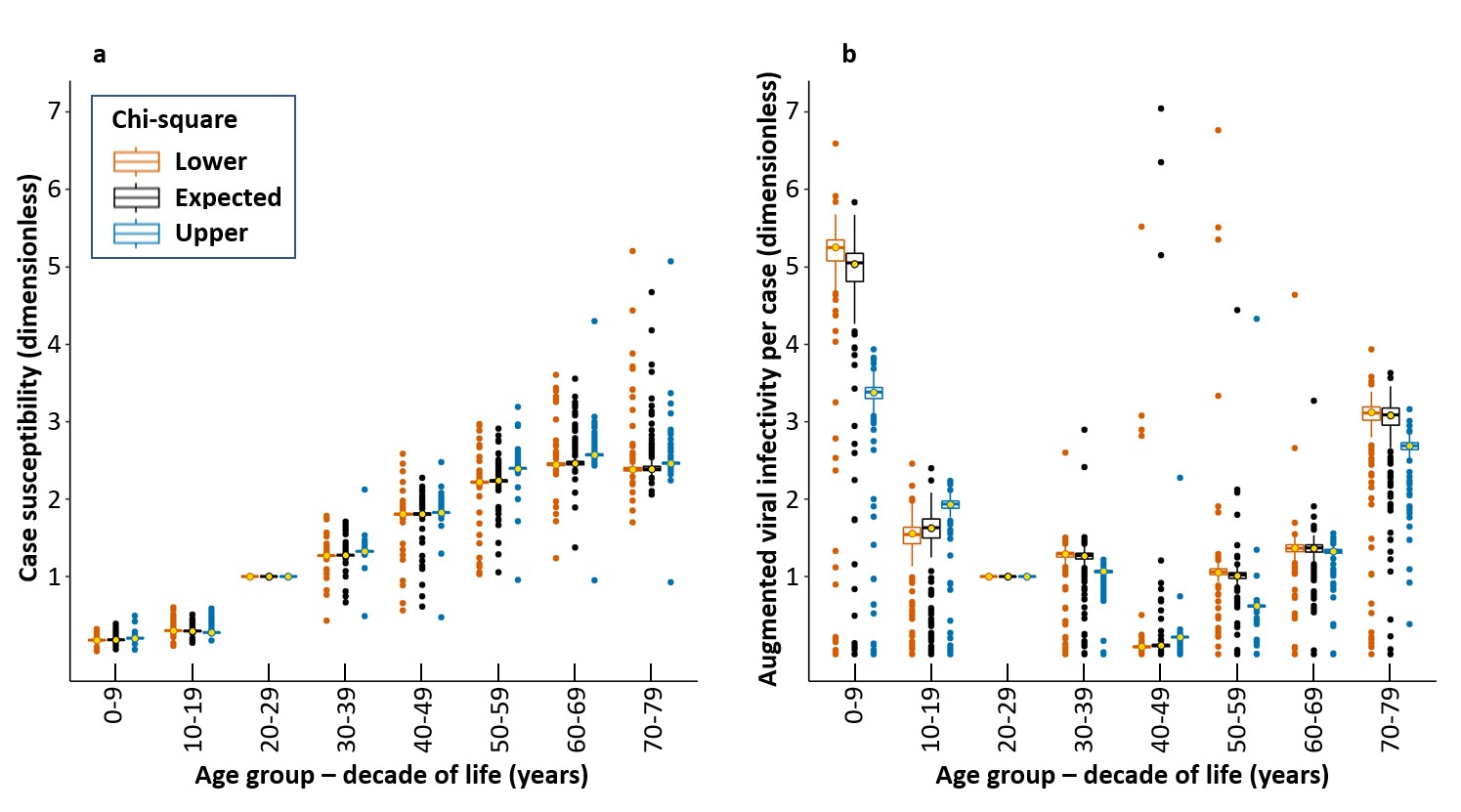


**Fig S1. Boxplots of case susceptibility and augmented infectivity per case from 201 basin-hopping runs.**

Fig S1 estimates the sensitivity of the regularized fit in Fig 1 to the smoothness imposed on it. The format of Fig S1 is like the format of Fig 1. Within each of the 8 age-groups, decades of life 0-9, 10-19, …, 70-79, the black points in the middle are identical to the ones in Fig 1 for $\chi^{2}$=42; the orange points on the left correspond to corresponds to $\chi^{2}$=42-√84; the blue points on the right, to $\chi^{2}$=42+√84.

# Discussion

Our methods maximized a regularized chi-square approximation related to log maximum likelihood. We recommend, however, that future analysis use regularized Bayesian statistics, which do not suffer the many statistical inadequacies of the maximum likelihood methods in the present analysis.

In the ground state of epidemiological ignorance, subclinical infections do not contribute to case counts, but the properties of infectious spread are otherwise similar. Linear analysis of the exponential phase produces a similarity transformation for $\tilde{\mathbf{R}}=\tilde{\mathbf{P}}\mathbf{R}\tilde{\mathbf{P}}^{-1}$ and $\tilde{\mathbf{T}}=\tilde{\mathbf{P}}\mathbf{T}\tilde{\mathbf{P}}^{-1}$ that confounds estimation of the matrix of transmission per contact and the NGM (see Eqs (5) and (6); also, Figs 2 and 3). Fortunately, the matrix $\tilde{\mathbf{P}}$ defining the similarity is a diagonal matrix, with age-stratified clinical fractions along its diagonal and off-diagonal elements 0. It therefore leaves the diagonals of $\tilde{\mathbf{R}}=\tilde{\mathbf{P}}\mathbf{R}\tilde{\mathbf{P}}^{-1}$ and $\tilde{\mathbf{T}}=\tilde{\mathbf{P}}\mathbf{T}\tilde{\mathbf{P}}^{-1}$ (the parameters pertinent to within-group spread) unchanged. In the presence of covert infections, similarity transformations are likely to be a feature of many mathematical analyses, particularly if the corresponding models mirror a ground state for epidemiological ignorance and do not distinguish between infectious properties of subclinical infections and cases.

# References

Asmussen, S., 2003. Applied Probability and Queues. Springer-Verlag, New York.

Backer, J.A., Klinkenberg, D., Wallinga, J., 2020. Incubation period of 2019 novel coronavirus (2019-nCoV) infections among travellers from Wuhan, China, 20-28 January 2020. Euro Surveill 25.

Davies, N.G., Klepac, P., Liu, Y., Prem, K., Jit, M., Eggo, R.M., 2020. Age-dependent effects in the transmission and control of COVID-19 epidemics. Nat Med 26, 1205-1211.

Diekmann, O., Heesterbeek, H., Britton, T., 2013. Mathematical Tools for Understanding Infectious Disease Dynamics. Princeton University Press.

Diekmann, O., Heesterbeek, J.A.P., Metz, J.A.J., 1990. On the Definition and the Computation of the Basic Reproduction Ratio R0 in Models for Infectious-Diseases in Heterogeneous Populations. Journal of Mathematical Biology 28, 365-382.

Diekmann, O., Heesterbeek, J.A.P., Roberts, M.G., 2010. The construction of next-generation matrices for compartmental epidemic models. Journal of the Royal Society Interface 7, 873-885.

Fischer, S.M., Lewis, M.A., 2021. A robust and efficient algorithm to find profile likelihood confidence intervals. Statistics and Computing 31, 38.

Ganyani, T., Kremer, C., Chen, D., Torneri, A., Faes, C., Wallinga, J., Hens, N., 2020. Estimating the generation interval for coronavirus disease (COVID-19) based on symptom onset data, March 2020. Euro Surveill 25, 2000257.

Harris, J.D., Park, S.W., Dushoff, J., Weitz, J.S., 2023. How time-scale differences in asymptomatic and symptomatic transmission shape SARS-CoV-2 outbreak dynamics. Epidemics 42, 100664.

Kucharski, A.J., Russell, T.W., Diamond, C., Liu, Y., Edmunds, J., Funk, S., Eggo, R.M., 2020. Early dynamics of transmission and control of COVID-19: a mathematical modelling study. Lancet Infect Dis 20, 553-558.

Lancaster, P., Tismenetsky, M., 1985. The Theory of Matrices. Academic Press, New York.

Nishiura, H., Chowell, G., Safan, M., Castillo-Chavez, C., 2010. Pros and cons of estimating the reproduction number from early epidemic growth rate of influenza A (H1N1) 2009. Theor. Biol. Med. Model. 7, 1.

Prem, K., Cook, A.R., Jit, M., 2017. Projecting social contact matrices in 152 countries using contact surveys and demographic data. PLoS Comput Biol 13, e1005697.

Press, W.H., Teukolsky, S.A., Vetterling, W.T., Flannery, B.P., 1976. Numerical Recipes in C, 2 ed. Cambridge University Press, Cambridge.

Rai, B., Shukla, A., Dwivedi, L.K., 2021. Estimates of serial interval for COVID-19: A systematic review and meta-analysis. Clin Epidemiol Glob Health 9, 157-161.

Seneta, E., 1981. Non-negative matrices and Markov chains. Springer-Verlag, New York.

Spouge, J.L., 2021. A comprehensive estimation of country-level basic reproduction numbers R0 for COVID-19: Regime regression can automatically estimate the end of the exponential phase in epidemic data. PLOS ONE 16, e0254145.

van den Driessche, P., Watmough, J., 2002. Reproduction numbers and sub-threshold endemic equilibria for compartmental models of disease transmission. Math. Biosci. 180, 29-48.

Wallinga, J., Lipsitch, M., 2007. How generation intervals shape the relationship between growth rates and reproductive numbers. Proc Biol Sci 274, 599-604.
